# Metabolic engineering of *Escherichia coli* for the biosynthesis of alpha-pinene

**DOI:** 10.1186/1754-6834-6-60

**Published:** 2013-04-30

**Authors:** Jianming Yang, Qingjuan Nie, Meng Ren, Hongru Feng, Xinglin Jiang, Yanning Zheng, Min Liu, Haibo Zhang, Mo Xian

**Affiliations:** 1 Qingdao Institute of Bioenergy and Bioprocess Technology, Chinese Academy of Sciences, Qingdao, 266101, China; 2 Foreign Languages School, Qingdao Agricultural University, Qingdao, 266109, China; 3 School of Life and Science, Nanjing Agricultural University, Nanjing, 210095, China; 4 College of Bioscience and Biotechnology, Shenyang Agricultural University, Shenyang, 110866, China; 5 College of Chemical Engineering and Technology, Wuhan University of Science and Technology, Wuhan, 430081, China

**Keywords:** α-Pinene, Geranyl diphosphate synthase, α-Pinene Synthase, *E*. *coli*

## Abstract

**Background:**

α-Pinene is an important natural product that is widely used in flavorings, fragrances, medicines, fine chemicals and high-density renewable fuels. Currently, α-Pinene used in industry is mainly produced either by tapping trees (gum turpentine) or as a byproduct of paper pulping (crude sulfate turpentine, CST). However, the extraction of it from trees is tedious and inefficient and requires substantial expenditure of natural resources. Therefore, it is necessary to seek sustainable technologies for α-pinene production.

**Results:**

To construct the microbial synthetic pathway of α-pinene in *E*. *coli*, we co-expressed native geranyl diphosphate synthase (IspA) from *E*. *coli* and α-pinene synthase (Pt30) from *Pinus taeda*, and then to increase the geranyl diphosphate (GPP) content in the cells, a suitable geranyl diphosphate synthase (GPPS2) was selected from two different origins. Furthermore, to enhance α-pinene production, a novel biosynthetic pathway of α-pinene was assembled in *E*. *coli* BL21(DE3) with the heterologous hybrid mevalonate (MVA) pathway, GPPS2 and α-pinene synthase (Pt30). The final genetic strain, YJM28, harboring the above novel biosynthetic pathway of α-pinene, accumulated α-pinene up to 5.44 mg/L and 0.97 g/L under flask and fed-batch fermentation conditions, respectively. The conversion efficiency of glucose to α-pinene (gram to gram) in the metabolically engineered strain reached 2.61%.

**Conclusions:**

In this paper, by using metabolic engineering techniques, the more efficient biosynthetic pathway of α-pinene was successfully assembled in *E*. *coli* BL21(DE3) with the heterologous hybrid MVA pathway, GPPS2 and α-pinene synthase (Pt30). In addition, this is the first report on α-pinene fed-batch fermentation, and our results represent improvements over previous reports.

## Background

α-Pinene is a natural and active monoterpene, which is derived from the head-to-tail condensation of dimethylallyl pyrophosphate (DMAPP) and isopentenyl pyrophosphate (IPP) [1]. α-Pinene is an important natural product that is widely used in flavorings, fragrances [2], medicines and fine chemicals [3,4]. Because of its compact structure and reactive olefin functionality, it also has some potential as a feedstock for high-density renewable fuels such as jet fuel [5,6].

Monoterpenes such as α-pinene, Î²-pinene, camphene, and limonene are important components of pine resin and are currently produced on an industrial scale either by tapping trees (gum turpentine) or as a byproduct of paper pulping (crude sulfate turpentine, CST) [2]. However, the extraction of these molecules from plants is tedious and inefficient and requires substantial expenditure of natural resources because of their low content [7]. Consequently, there is much interest in developing technologies that could engineer microorganisms to convert renewable resources, such as glucose generated from cellulose or hemicellulose, into monoterpene products [8]. By comparison with traditional methods, microbial synthesis of α-pinene seems likely to become successful primarily because microorganisms have the advantages of fast growth, no need for land during their growth and sustainable development [9,10].

**Figure 1:**
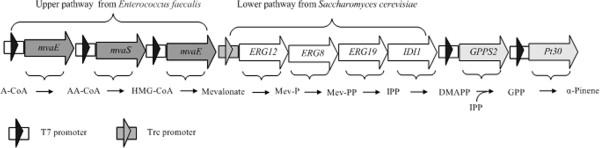
**Production of α-pinene via the MVA pathways used in this study.** Gene symbols and the enzymes they encode (all genes marked with gray arrows were from *Enterococcus faecalis*, all genes marked with white arrows were isolated from *Saccharomyces cerevisiae* and the gene marked with light gray arrows derived from *Abies grandis* or *Pinus taeda*). MVA pathway: MvaE, acetyl-CoA acetyltransferase /HMG- CoA reductase; MvaS, HMG-CoA synthase; ERG12, mevalonate kinase; ERG8, phosphomevalonate kinase; ERG19, mevalonate pyrophosphate decarboxylase; IDI1, IPP isomerase;GPPS2, *Abies grandis* geranyl diphosphate synthase was optimized to the preferred codon usage of *E. coli*; Pt30, *Pinus taeda* alpha-pinene synthase was optimized to the preferred codon usage of *E. coli*. Pathway intermediates. MVA pathway: A-CoA, acetyl-CoA; AA-CoA, acetoacetyl-CoA; HMG-CoA, hydroxymethylglutaryl-CoA; Mev-P, mevalonate 5-phosphate; Mev-PP, mevalonate pyrophosphate. IPP, isopentenyl pyrophosphate; DMAPP, dimethylallyl pyrophosphate; GPP, geranyl diphosphate.

Like other monoterpenes, α-pinene is generated from the common precursor of IPP and DMAPP, which can be produced from two different pathways: the methylerythritol 4-phosphate (MEP) pathway and the mevalonate (MVA) pathway (Figure 1) [11]. Although many microorganisms carry out the MEP pathway or MVA pathway to supply the intermediates DMAPP and IPP, they are unable to produce the monoterpenes because of the absence of monoterpene synthase. With the substantial improvement of metabolic engineering, many research labs have explored biosynthetic methods of monoterpene production by introducing heterologous monoterpene synthase: for instance, 3-carene and limonene. Reiling *et al*. demonstrated that an engineered *E*. *coli* strain that overexpresses native 1-deoxy-D-xylulose-5-phosphate (DXP) synthase (DXS) and IPP isomerase (IPIHp) from *Haematococcus pluvialis*, geranyl diphosphate synthase (IspA) from *E*. *coli* and 3-carene cyclase from *Picea abies* can accumulate a 3-carene titer of about 3 μg/L/OD_600_ after 8 h production [12]. By using the native MEP pathway to supply the precursor of DMAPP and IPP, Carter *et*.*al*. introduced a monoterpene biosynthesis pathway into *E*. *coli*, which led to about 5 mg/L limonene production [13].

Although great success has been achieved in monoterpene production by using the MEP pathway, this approach remains inefficient owing to regulatory mechanisms present in the native host [14]. This limitation was also confirmed by experiments on isoprene production using the MEP or MVA pathway [15,16].

## Results and discussion

In this paper, α-pinene production was significantly enhanced by assembling a biosynthetic pathway of α-pinene using the heterologous MVA pathway and the *GPPS2* and *Pt30* genes in an engineered *E*.*coli* strain. The final genetic strain, YJM28, containing the biosynthetic pathway of α-pinene, accumulated α-pinene up to 5.44 mg/L and 0.97 g/L under flask and fed-batch fermentation conditions, respectively. The conversion efficiency of glucose to α-pinene (gram to gram) reached 2.61%. To our knowledge, this is the first report on α-pinene fed-batch fermentation, and our results represent improvements over previous reports. Thus, an alternative production system for α-pinene from renewable sources via the MVA pathway in *E*. *coli* has been provided.

### Characterization of α-pinene by GC-MS

**Figure 2:**
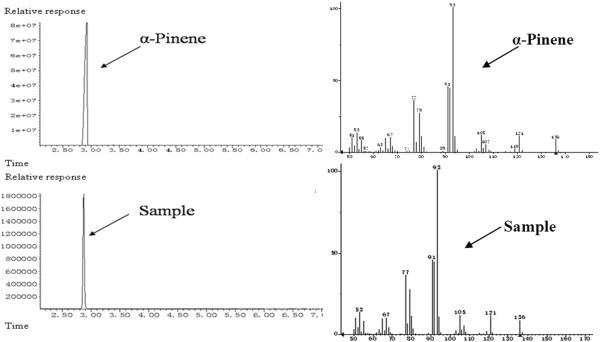
**GC-MS analysis of sample from the headspace of the sealed cultures of strain YJM26**. When OD_600_ reached 0.6-0.9, cultures were induced at 30°C for 24 h using 1 mM IPTG.

Although *E*. *coli* possesses a native MEP pathway that supplies the intermediates DMAPP and IPP, it cannot produce α-pinene because of the absence of α-pinene synthase. Consequently, to synthesize α-pinene, α-pinene synthase (Pt30) derived from *Pinus taeda* was introduced into the *E*. *coli* strain. However, after 40 h of incubation of the modified strain, the target product could not be measured by GC-MS (data not shown). The main reason for this result might be the failure to detect α-pinene since its production was too low as a result of the insufficiency of GPP. Hence, to enhance the supply of GPP, the native gene *ispA* from *E*. *coli*, which encodes geranyl diphosphate synthase, which catalyzes the conversion of DMAPP and IPP into GPP, was combined with the α-pinene synthase gene (*Pt30*) and ligated into pACYDUet-1 to create the plasmid pYJM23 (pACY-ispA-Pt30). The *E*. *coli* strain containing pYJM23 was inoculated in the fermentation medium and incubated at 37°C with shaking at 180 rpm. When its OD_600_ (cell culture optical density measured at 600 nm , one OD_600_ unit approximately corresponded to 0.43 g L^-1^ of dry cell weight) reached 0.6, IPTG was added to a final concentration of 1 mM, and culture was further maintained at 30°C for 24 h. The off-gas from the headspace of the sealed cultures was tested by GC-MS. As shown in Figure 2, based on the relative retention time and total ion mass spectral comparison with an external standard, the engineered *E*. *coli* strain carrying the native *ispA* gene and *Pt30* from *Pinus taeda* produced α-pinene in detectable quantities. Thus, the biosynthetic pathway for α-pinene production was successfully constructed using the MEP pathway and *Pt30* from *Pinus taeda*.

### Screening of geranyl diphosphate synthases

As an important metabolic precursor of the monoterpenes, geranyl diphosphate (GPP) is generated from the condensation of dimethylallyl diphosphate and isopentenyl diphosphate, catalyzed by GPPS, which is regarded as a short-chain prenyltransferase that functions at the branch points of isoprenoid metabolism [1]. An effective method to optimize pathway efficiency may be to use genes from different organisms [17]. In this study, the GPPS enzymes from *E*. *coli* and *Abies grandis* were evaluated to enhance the supply of GPP.

Because of the difficulty in detecting and quantifying GPP, the *GPPS2* gene from *Abies grandis* or *ispA* gene from *E*. *coli* was ligated with the α-pinene synthase gene (*Pt30*) to create the plasmid pYJM25(pACY-GPPS2-Pt30)or pYJM23 (pACY-ispA-Pt30), respectively. The strains YJM26 (carrying pYJM23), YJM27 (carrying pYJM25), and YJM29(carrying pYJM28) were cultured in 100-ml shake-flasks. When each culture reached an OD_600_ of 0.6, expression of GPP synthase and α-pinene synthase was induced by 0.5 mM IPTG. Meanwhile, 200 μM DMAPP and IPP were added into the medium, and the culture was further incubated at 30°C for 24 h. A noticeable difference in α-pinene production was observed. The *E*. *coli* strain YJM27 harboring *GPPS2* and *Pt30* genes produced 1.35 mg α-pinene per liter of bacterial culture, which was about 8-fold higher than strain YJM26 harboring *ispA* and *Pt30* genes (0.172 mg/L), whereas the *E*. *coli* strain(YJM29)carrying only the α-pinene synthase generated no detectable α-pinene. This result demonstrates that the exogenous expression of geranyl diphosphate synthase contributed to the α-pinene production, and the enzyme activity of GPPS2 from *Abies grandis* was higher than that of IspA from *E*. *coli* BL21(DE3). Hence, the GPPS2 enzyme was selected to enhance GPP production in the following experiments.

### Biosynthesis of α-pinene using the MVA pathway

In previous experiments, a hybrid exogenous MVA pathway has been assembled in engineered *E*.*coli* strains to produce isoprene [15]. Based on previous experimental data, the hybrid exogenous MVA pathway is effective to synthesize DMAPP and IPP. Consequently, we hypothesized that the engineered strain with the hybrid exogenous MVA pathway could further enhance the production of α-pinene.

To test the effect of the MVA pathway on the production of α-pinene, the recombinant strain YJM28 (*E*. *coli* harboring the MVA pathway, GPPS and α-pinene synthase) and strain YJM27 (*E*. *coli* harboring GPPS synthase and α-pinene synthase) were cultured in fermentation medium under shake-flask conditions. The amount of α-pinene accumulated in the culture media from different recombinant strains was calculated according to a standard curve plotted with a set of known concentrations of α-pinene. The α-pinene concentration of strain YJM28 reached 0.65 mg/L after being induced by 0.5 mM IPTG for 24 h, while strain YJM27 (without the MVA pathway) produced only a small amount of end product (data not shown). These results indicate that the hybrid MVA pathway caused a considerable increase in α-pinene production.

### Optimization of fermentation conditions

Fermentation conditions are of great importance because they play a vital role in the formation, concentration and yield of end product [18]. Optimizing fermentation conditions for strains is a good way to improve product quality and quantity.

The one-factor-at-a-time method is a closed-ended system for fermentation processes. Based on the classical method of changing one independent variable while fixing all others [19,20], this method can be applied to optimization of medium components as well as to optimization of process conditions. In view of its easy operation and convenience, the one-factor-at-a-time method has become the most popular method for improving fermentation media and process conditions. In this paper, the three most important factors, induction temperature, inducer concentration and organic nitrogen source, were optimized to improve α-pinene production, using the strain YJM28.

#### *Effect of induction temperature on α-pinene production*

**Figure 3:**
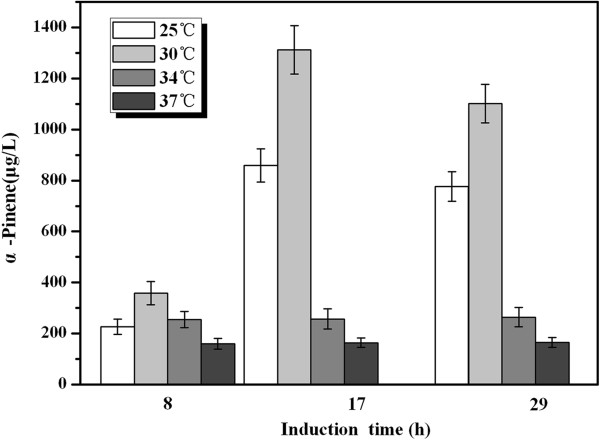
**Effect of induction temperature on α-pinene production by YJM28.** When OD_600_ reached 0.6-0.9, cultures were induced at different temperatures: 25°C (white color), 30°C (light-gray color), 34°C (gray color), and 37°C (dark-gray color) for 29 h using 1 mM IPTG. The experiment was conducted in triplicate.

Low induction temperatures (30°C or 25°C) can enhance the activities of recombinant enzymes because low temperatures decrease the inclusion bodies in genetically engineered *E*. *coli*[21,22]. However, a successful control of cultivation temperature has to balance the enzyme expression, cell growth and product formation [23]. Hence, in this study, to increase α-pinene production, the induction temperatures of 25°C, 30°C, 34°C, and 37°C were tried. As shown in Figure 3, the maximum α-pinene production was observed at 30°C, at 1.31 mg/L, which was about 1.5 times, 5 times and 8 times greater than those observed at 25°C (0.86 mg/L), 34°C (0.26 mg/L) and 37°C (0.165 mg/L), respectively. Hence, the optimum induction temperature for α-pinene production was 30°C.

#### *Effect of inducer concentration on α-pinene production*

**Figure 4:**
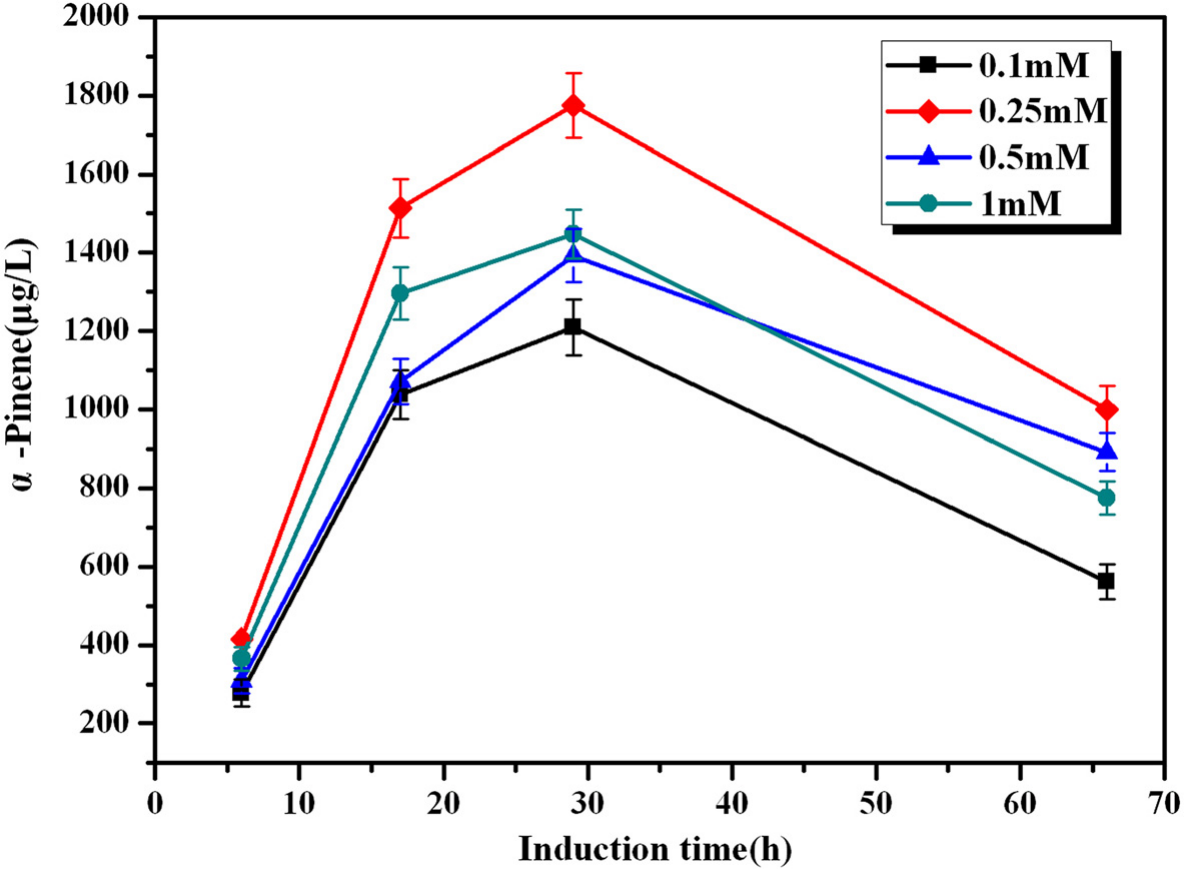
**Effect of inducer concentration on α-pinene production by YJM28**. When OD_600_ reached 0.6-0.9, cultures were induced at 30°C for 66 h using different concentrations of IPTG: 0.1 mM (■), 0.25 mM (♦), 0.5 mM (▲), 1 mM (●) The experiment was conducted in triplicate.

Exogenous gene expression generally causes a metabolic burden on the cell, which can result in reduced growth rates, cell yields, product expression, and plasmid stability [24,25]. The level of IPTG used can be varied to adjust the extent of the metabolic burden imposed on the cell [26]. Based on these considerations, various IPTG concentrations, ranging from 0.1 mM to 1 mM, were tested to optimize the inducer concentration at the above-optimized temperature 30°C. According to the data shown in Figure 4, the production of α-pinene reached a maximum of 1.77 mg/L at the IPTG concentration of 0.25 mM.

#### *Effect of organic nitrogen source on α-pinene production*

**Figure 5:**
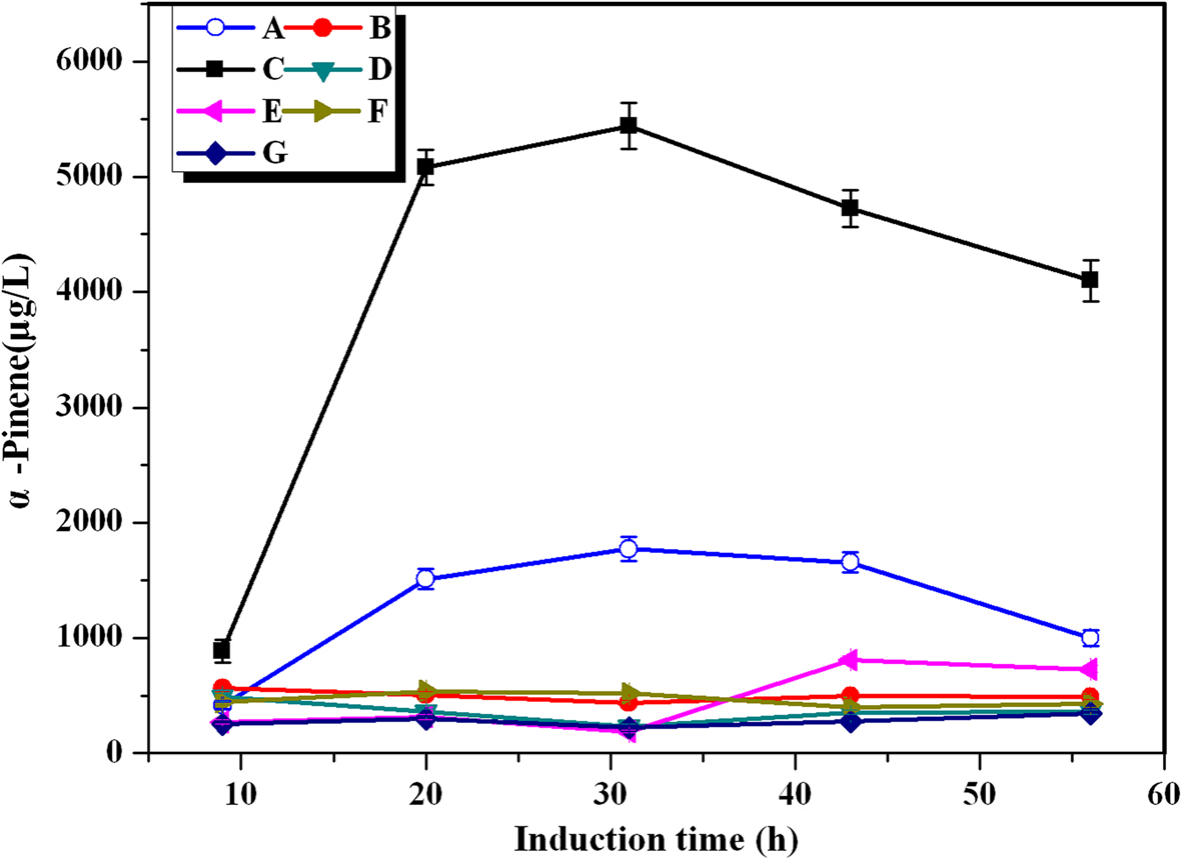
**Effect of different organic nitrogen source on α-pinene production by YJM28.** A: beef extract (Aladdin, ○); B: beef extract (Beijing AoBoXing Bio-Tech Co., Ltd, ●); C: beef extract powder (MDBio, Inc, ■); D: beef extract powder (Beijing AoBoXing Bio-Tech Co., Ltd, ▼); E: beef extract (Beijing Shuangxuan Microbe Culture Medium Products Factory, ◄); F: beef extract (Sinopharm Chemical Reagent Co., Ltd, ►); G: beef extract (solarbio, ♦). When OD_600_ reached 0.6-0.9, cultures were induced at 30°C for 56 h using 0.25 mM IPTG. The experiment was performed in triplicate.

The source of the nitrogen in the medium plays an important role in improving the biosynthesis of desired product [27]. To investigate the effect of organic nitrogen source on α-pinene production, seven different organic nitrogen sources were assessed at the above-optimized temperature and IPTG concentration (Figure 5). Among the organic nitrogen supplements tried, the MD beef extract permitted a significantly higher α-pinene production than the other organic nitrogen sources. The highest concentration of α-pinene was 5.44 mg/L, which was about 16 times as much as the lowest observed. This productivity was higher than the highest previously reported [28].

Based on the above data, the most suitable culture conditions for α-pinene production using the engineered strain YJM28 were 30°C, 0.25 mM IPTG and MD beef extract as the organic nitrogen source.

### Fed-batch culture of the engineered strains

**Figure 6:**
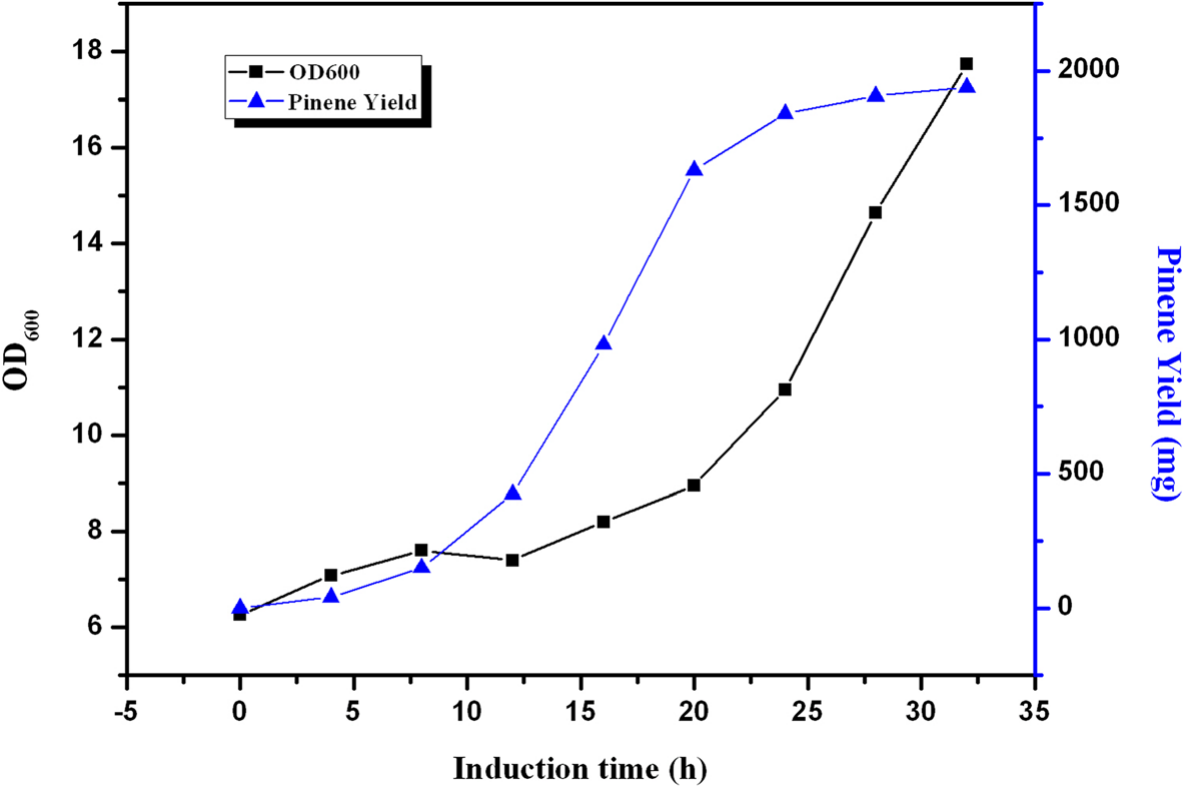
**The time course of α-pinene production by YJM28**. α-Pinene accumulation (▲) and cell growth (■) in YJM28. Induction was carried out at 12 h at 30°C and 0.25mM IPTG. Other experimental conditions are described in section “Fed-Batch Fermentation”.

To further determine the ability of the engineered strain to produce α-pinene at high yield, fed-batch fermentation was carried out using the engineered *E*. *coli* BL21(DE3) strain simultaneously harboring plasmids pYJM27(pACY-mvaE-mvaS- GPPS2-Pt30) and pYJM14(pTrc-ERG12-ERG8-ERG19-IDI1). During the fed-batch operation, glucose was added intermittently when glucose became depleted, which was indicated by the sharp rise of pH. By controlling the concentration of acetic acid, which is the most deleterious by-product during the culture process of *E*. *coli*, we maintained the glucose concentration during the fed-batch culture at less than 0.5 g/L. As shown in Figure 6, α-pinene production increased rapidly from 8 h to 20 h after induction, and the specific productivity reached 0.018 g h^-1^g^-1^ dry cells. After the cultures were induced for 32 h, α-pinene accumulated and reached a maximum concentration of 0.97 g L^-1^ with a specific productivity of 0.004 gh^-1^ g^-1^ dry cells, and the conversion efficiency of glucose to α-pinene (gram to gram) reached 2.61%. However, the cell density of the engineered strain reached only about 18, which was rather low for the fed-batch fermentation of *E*. *coli* strains. The main reason for the low cell mass of *E*. *coli* strain may lie in the retardation of cell growth resulting from overexpression of many heterologous genes. In the previous studies, other monoterpene has been reported to be produced. For example, Carter *et al*. demonstrated the production of limonene in engineered *E*. *coli* strain with co-expression of GPP synthase, IPP isomerase and limonene synthase, which could achieve production levels of 5 mg/L in 24-h culture runs [13]. It is difficult to compare these results to ours primarily because of the following two reasons: one is the different pathways that were used, that is DXP pathway for limonene production and MVA pathway for α-pinene synthesis; the other is that they utilized quite different growth and production conditions and strains.

Despite the extensive progress made on α-pinene production in *E*. *coli*, many possible improvements can be achieved to enhance α-pinene production. One approach is to optimize the fermentation process by increasing cell density to elevate the yield of product [29,30]. Another possibility is employing a chromosome integration technique to decrease the cell growth burden on the host that results from overexpression of heterologous genes [31,32].

## Conclusions

In this study, α-pinene production was significantly enhanced by assembling a biosynthetic pathway of α-pinene using the heterologous MVA pathway and the *GPPS2* and *Pt30* genes in an engineered *E*.*coli* strain. The final strain, YJM28, containing the biosynthetic pathway of α-pinene, accumulated α-pinene up to 5.44 mg/L and 0.97 g/L under the flask and fed-batch fermentation conditions, respectively. The conversion efficiency of glucose to α-pinene (gram to gram) in the metabolically engineered strain reached 2.61%. To our knowledge, this is the first report on α-pinene fed-batch fermentation, and our results represent improvements over previous reports. Therefore, an alternative production system for α-pinene from renewable sources via the MVA pathway in *E*. *coli* has been provided.

## Methods

### Bacterial strains, plasmids, and growth conditions

All strains and plasmids used in this study are listed in Table 1. *E*. *coli* strains were grown in LB medium. For α-pinene production, the different strains were cultivated in shake-flask or fed-batch fermentation conditions with medium containing glucose 20 g/L, K_2_HPO_4_ 9.8 g/L, beef extract 5 g/L, ferric ammonium citrate 0.3 g/L, citric acid monohydrate 2.1 g/L, MgSO_4_ 0.06 g/L and 1 ml trace element solution, which included (NH_4_)_6_Mo_7_O_24_·4H_2_O 0.37 g/L, ZnSO_4_·7H_2_O 0.29 g/L, H_3_BO_4_ 2.47 g/L, CuSO_4_·5H_2_O 0.25 g/L, and MnCl_2_·4H_2_O 1.58 g/L. If necessary, appropriate antibiotics were added to the culture medium at the following concentrations: ampicillin (Amp, 100 μg/ml) and chloramphenicol (Cm, 34 μg/ml).

### Plasmid construction

Standard DNA manipulations were executed as previously described [33]. Polymerase chain reaction (PCR) was performed using *Pfu* DNA polymerase (TaKaRa, Dalian, China) according to the manufacturer’s instructions.

### Construction of plasmids for GPP synthase screening

**Figure 7:**
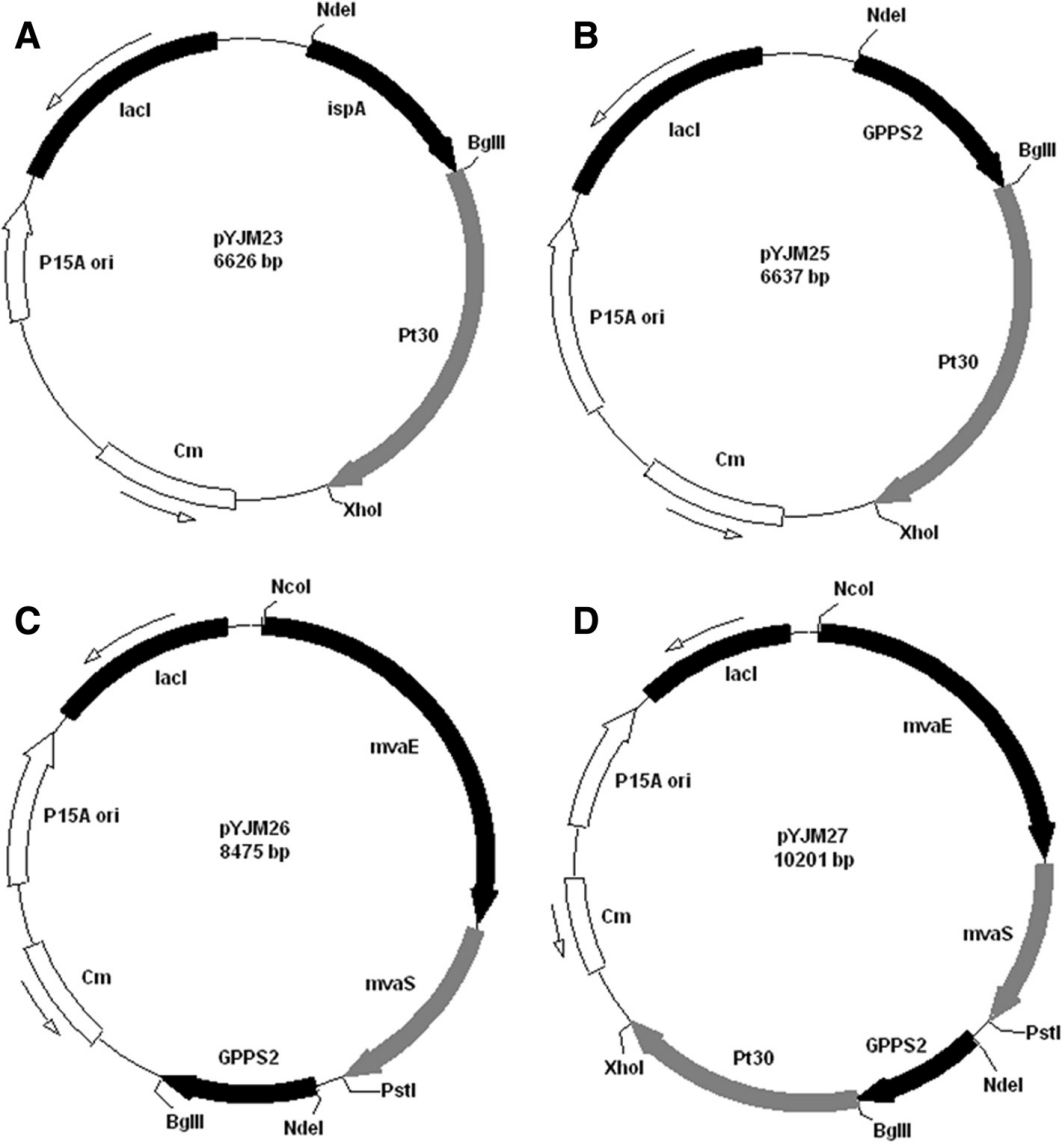
**Plasmids used in this study.** (**A**) represented the plasmid pYJM23 harboring *ispA* from *E. coli* BL21(DE3) and *Pt30* from *Pinus taeda*; (**B**) represented the plasmid pYJM25 carrying *GPPS2* from *Abies grandis* and *Pt30* from *Pinus taeda*; (**C**) represented the plasmid pYJM26 containing *mvaE and mvaS* from *Enterococcus faecalis, GPPS2* from *Abies grandis*; (**D**) represented the plasmid pYJM27 carrying *mvaE and mvaS* from *Enterococcus faecalis, GPPS2* from *Abies grandis*, *Pt30* from *Pinus taeda.*

The *IspA* gene was obtained by PCR using the primers IspA-F (5’-GGGAATTCC- ATATGATGGACTTTCCGCAGCAACTC-3’) and IspA-R (5’-GGAAGATCTTT ATTTA-TTACGCTGGATGATGT-3’) and *E*. *coli* BL21(DE3) genomic DNA as a template. The isolated *IspA* gene fragment was excised using BglII and NdeI, followed by insertion into the corresponding sites of the vector pACYCDuet-1 to create pYJM22. The *Pt30* gene fragment was acquired by excision from pGH/Pt30 with BglII and XhoI and was introduced into the corresponding sites of pYJM22 to create pYJM23 (Figure 7A).

**Table 1:** Strains and plasmids used in this study

**Name**	**Relevant characteristics**	**References**
**Strains**		
*E.coli* BL21(DE3)	F^-^*ompT hsd*S_B_ (r_B_^-^m_B_^-^) *gal dcm rne*131 Î»(DE3)	Invitrogen
*Saccharomyces cerevisiae*	Type strain	ATCC 4040002
YJM26	*E.coli* BL21(DE3)/pYJM23	This study
YJM27	*E.coli* BL21(DE3)/pYJM25	This study
YJM28	*E.coli* BL21(DE3)/pYJM27,pYJM14	This study
YJM29	*E.coli* BL21(DE3)/pYJM28	This study
**Plasmids**		
pACYCDuet-1	P15A (pACYC184), Cm^r^	Novagen
pTrcHis2B	pBR322 origin, Amp^r^	Invitrogen
pYJM14	pTrcHis2B carrying *ERG12*, *ERG8*, *ERG19* and *IDI1* from *Saccharomyces cerevisiae*	(Yang et al., 2011) [35]
pYJM22	pACYCDuet-1 carrying *ispA* from *E.coli* BL21(DE3)	This study
pYJM23	pACYCDuet-1 carrying *ispA* from *E.coli* BL21(DE3) and *Pt30* from *Pinus taeda*	This study
pYJM24	pACYCDuet-1 carrying *GPPS2* from *Abies grandis*	This study
pYJM25	pACYCDuet-1 carrying *GPPS2* from *Abies grandis* and *Pt30* from *Pinus taeda*	This study
pYJM26	pACYCDuet-1 carrying *mvaE and mvaS* from *Enterococcus faecalis, GPPS2* from *Abies grandis*	This study
pYJM27	pACYCDuet-1 carrying *mvaE and mvaS* from *Enterococcus faecalis, GPPS2* from *Abies grandis*, *Pt30* from *Pinus taeda*	This study
pYJM28	pACYCDuet-1 carrying *Pt30* from *Pinus taeda*	This study

The geranyl diphosphate synthase (*GPPS2*) gene (GenBank No. AF513112) from *Abies grandis* and alpha-pinene synthase (*Pt30*) gene (GenBank No. AF543530) from *Pinus taeda* were analyzed by online software (http://www.genscript.com/cgi-bin/tools/rare_codon_analysis) and optimized to the preferred codon usage of *E*. *coli* (http://www.jcat.de/). The codon-optimized *GPPS2* gene and *Pt30* gene were synthesized by Genray Company with plasmid pGH as the vector (named pGH/*GPPS2* and pGH/*Pt30*, respectively). The *GPPS2* gene fragment was obtained by digestion of pGH/GPPS2 with NdeI and BglII and then ligated into the corresponding sites of pACYCDuet-1 to create pYJM24. The *Pt30* gene fragment was acquired by excision of pGH/Pt30 with BglII and XhoI and was introduced into the corresponding sites of pYJM24 to create pYJM25 (Figure 7B).

### Construction of plasmids for the whole pathway of α-pinene synthesis

The *mvaE*-*mvaS* gene fragment was obtained by excision from pYJM20 [15] using NcoI and PstI and then ligated into the corresponding sites of pYJM24 to create pYJM26 (Figure 7C). The *Pt30* gene fragment was acquired by excision from pGH/Pt30 with BglII and XhoI and was introduced into the corresponding sites of pYJM26 to create pYJM27 (Figure 7D).

Plasmid pYJM14 was constructed based on pTrcHis2B by introducing *ERG8*, *ERG12*, *ERG19* and *IDI1* from *S*. *cerevisiae*[34].

### α-Pinene characterization by GC-MS

Putative α-pinene products were analyzed by capillary GC-MS using an Agilent 5975C System chromatograph equipped with an HP-INNOWAX capillary column (30 m×0.25 mm×0.25 μm, Agilent, Palo Alto, CA, USA). The separation conditions were an initial column temperature of 40°C for 1 min, an increase of 4°C/min to 70°C and an increase of 25°C/min to 300°C, where it was held for 5 min. The other parameters were an injector temperature of 250°C; ion source temperature 230°C; EI 70 eV; carrier gas He at a flow rate of 1 ml min^-1^; split ratio 1:7; mass range 35-300 m/z. Peak identification was based on a relative retention time and total ion mass spectral comparison with an external standard.

### α-Pinene quantification by gas chromatography (GC)

The α-pinene produced by the recombinant strains was quantitatively analyzed by GC as described previously [35]. The *E*. *coli* strain was inoculated in 50 ml of fermentation medium containing 34 μg/ml chloramphenicol and/or 100 μg/ml ampicillin and then cultured at 37°C with shaking at 180 rpm. When the OD_600_ of the bacterial culture reached 0.6, the cells were induced by IPTG at a final concentration of 1 mM for 24 h. Then, the off-gas samples were taken from the headspace of the sealed cultures and analyzed by GC.

GC analysis was performed on an Agilent 7890A equipped with a flame ionization detector (FID) and an HP-INNOWAX column (25 m×250 μm×0.2 μm). N_2_ was used as carrier gas with a linear velocity of 1 ml/min. The column temperature profile was 50°C for 0.5 min, 4°C/min increase to 70°C, 25°C/min increase to 250°C, and 250°C for 5 min. The product was characterized by direct comparison with an authentic standard (Sigma-Aldrich). The peak area was converted into α-pinene concentration in comparison with a standard curve plotted with a set of known concentrations of α-pinene.

### Shake-flask cultures

Shake-flask experiments were performed in triplicate series of 600 ml sealed shake flasks containing 50 ml fermentation medium as described above plus 34 μg/ml Cm and 100 μg/ml Amp. *E*. *coli* strains were cultured in the broth and incubated in a gyratory shaker incubator at 37°C and 180 rpm. When the OD_600_ reached 0.6, IPTG was added to final concentration of 0.25 mM, and the culture was further incubated at 30°C for 24 h. Then, 1 ml of gas sample from the headspace of the sealed cultures was analyzed as described previously [35] using a gas chromatograph (Agilent 7890A, America) equipped with a FID and an HP-INNOWAX column (25 m×250 μm×0.2 μm). Concentrations of α-pinene synthesized by bacterial cells were calculated by converting the GC peak area into milligrams of α-pinene via a calibration curve.

### Optimization of fermentation process

#### Effect of induction temperature

The *E*. *coli* strain was inoculated in 50 ml of fermentation medium and cultured at 37°C with shaking at 180 rpm. When the OD_600_ of the bacterial culture reached 0.6-0.9, the shake-flask cultures were incubated at different induction temperatures (25°C, 30°C, 34°C or 37°C) for 29 h in 1 mM IPTG, and the α-pinene products were assayed.

#### Effect of IPTG concentration

The shake-flask culture was incubated in different inducer (IPTG) concentrations (0.1 mM, 0.25 mM, 0.5 mM or 1 mM) at the above-optimized temperature for 66 h, and the α-pinene products were measured.

#### Effect of organic nitrogen source

The shake-flask cultures were incubated in different organic nitrogen sources (beef extract (solarbio), beef extract (Aladdin), beef extract (Beijing Shuangxuan Microbe Culture Medium Products Factory), beef extract (Sinopharm Chemical Reagent Co., Ltd), beef extract powder (MDBio, Inc), beef extract (Beijing AoBoXing Bio-Tech Co., Ltd) or beef extract powder (Beijing AoBoXing Bio-Tech Co., Ltd)) at the above-optimized temperature and IPTG concentration for 56 h, and the pinene products were calculated.

### Fed-batch fermentation

The strain YJM28 was grown overnight at 37°C in 100 ml of LB medium (containing 10 g of NaCl, 5 g of yeast extract, and 10 g of tryptone per 1 L). These cultures were used to inoculate a 5-L fermentor (BIOSTAT Bplus MO5L, Sartorius, Germany) containing 2 L of fermentation medium. The temperature was maintained at 30°C, the pH was maintained at 7.0 via automated addition of ammonia, and Antifoam 204 was used to prohibit foam development. The stirring speed was first set at 400 rpm and then linked to the dissolved oxygen(DO) concentration to maintain a 20% saturation of DO. The expression of plasmid-born exogenous gene(s) for α-pinene production was initiated at an OD_600_ of 6 by adding IPTG at a final concentration of 0.25 mM, and IPTG was added every 8 h. During the course of fermentation, the residual glucose was measured using a glucose analyzer (SBA-40D, China) and maintained below 0.5 g/L by a feeding solution containing 800 g/L of glucose at appropriate rates. Then, α-pinene accumulation was measured every 60 min by GC as described in section Optimization of fermentation conditions. At the same time, the growth of the bacterial culture was determined by measuring the OD_600_ with a spectrophotometer (Cary 50 UV-vis, Varian).

### Calculations

The following equation was used to calculate the specific productivity [36].where Q_p_=specific production rate (g h^-1^g^-1^ dry cells); p=α-pinene concentration (g/L); t=cultivation time (h), and x=biomass (g/L).

$$Q_{p}=\frac{p_{1}-p_{0}}{t_{1}-t_{0}}\times \frac{2}{x_{1}+x_{0}}$$

Cell culture optical density was measured at 600 nm using a spectrophotometer (Cary 50UV-vis, VARIAN), and the dry cell weight was calculated according to the coefficient (one OD_600_ unit corresponded to 0.43 g L^-1^ of dry cell weight).

## Abbreviations

DMAPP: Dimethylallyl pyrophosphate; IPP: Isopentenyl pyrophosphate; GPP: Geranyl diphosphate; MVA: Mevalonate; MEP: Methylerythritol 4-phosphate; IPTG: Isopropyl Î²-D-thiogalactoside; PCR: Polymerase chain reaction; GC: Gas chromatography

## Competing interests

The authors declare that they have no competing interests.

## Authors' contributions

MX and JY developed the idea for the study. JYand HZ designed the research, did the literature review and prepared the manuscript. MX and QN helped to revise the manuscript. ML, YZ, XJ, HF and MR did the lab work, plasmid construction, strain cultivation, Fed-Batch Fermentation and product detection. All authors read and approved the final manuscript.
